# 4472 Plasma Neurofilament Light as a Biomarker for Pediatric Patients with Huntington’s Disease

**DOI:** 10.1017/cts.2020.310

**Published:** 2020-07-29

**Authors:** Jordan L Schultz

**Affiliations:** 1 University of Iowa Institute for Clinical and Translational Science

## Abstract

OBJECTIVES/GOALS: The goal of this study is to compare plasma neurofilament light (NfL) concentrations in asymptomatic children and young adults that carry the gene expansion (GE group) that causes Huntington’s Disease to similar subjects that do not carry this genetic mutation (GNE group). METHODS/STUDY POPULATION: Subjects from the Kids-HD study in the GE group were divided into groups based on their estimated years to motor onset. Each subgroup was compared to the subjects from the GNE group. Additionally, a group of participants with juvenile HD were compared to the GNE group. These comparisons were made by utilizing linear mixed effects regression models that included a random effect per subject and family and also included the covariates of age and parental socioeconomic status. A post-hoc analysis of subjects in the GE group who were within 20 years from their predicted motor onset was conducted to assess the relationship between striatal volume and plasma NfL concentrations. RESULTS/ANTICIPATED RESULTS: GE participants more than 20 years from their predicted motor onset did not have elevated plasma NfL concentrations relative to the GNE group. However, participants who were 15-20 years from their predicted motor onset had a mean NfL concentration of 1.61 pg/uL compared to 1.31 pg/uL in the GNE group (p = 0.036). Participants who were within 15 years from their predicted motor onset had a mean NfL concentration of 2.08 pg/uL, which was also significantly elevated relative to the GNE group (t = 3.03, p = 0.003). Additionally, the participants with juvenile HD had a mean NfL level of 3.22 pg/uL, which was significantly elevated compared to the GNE group (p<0.0001). NfL concentrations were significantly correlated with striatal volume amongst participants who were within 20 years of onset (p = 0.017). DISCUSSION/SIGNIFICANCE OF IMPACT: The huntingtin protein is essential to neurodevelopment but current gene therapies for HD focus on blocking production of this gene. These results will provide guidance on the optimal timing of administration of gene therapies by identifying neurodegeneration decades prior to motor onset of HD.

